# Elderly patients’ participation in emergency medical services when offered an alternative care pathway

**DOI:** 10.3402/qhw.v8i0.20014

**Published:** 2013-02-26

**Authors:** Veronica Vicente, Maaret Castren, Fredrik Sjöstrand, Birgitta Wireklint Sundström

**Affiliations:** 1 Section of Emergency Medicine, Department of Clinical Science and Education, Karolinska Institutet, Stockholm, Sweden; 2 School of Health Sciences, Research Centre PreHospen, The Prehospital Research Centre of Western Sweden, University of Borås, Borås, Sweden

**Keywords:** Caring, patient participation, lifeworld, lived experiences, choice of healthcare, geriatric, community-based hospital, suffering from care, caring encounter

## Abstract

As organizational changes in the healthcare system are in progress, to enhance care quality and reduce costs, it is important to investigate how these changes affect elderly patients’ experiences and their rights to participate in the choice of healthcare. The aim of this study is to describe elderly patients’ lived experience of participating in the choice of healthcare when being offered an alternative care pathway by the emergency medical services, when the individual patient's medical needs made this choice possible. This study was carried out from the perspective of caring science, and a phenomenological approach was applied, where data were analysed for meaning. Data consist of 11 semi-structured interviews with elderly patients who chose a healthcare pathway to a community-based hospital when they were offered an alternative level of healthcare. The findings show that the essence of the phenomenon is described as “There was a ray of hope about a caring encounter and about being treated like a unique human being”. Five meaningful constituents emerged in the descriptions: endurable waiting, speedy transference, a concerned encounter, trust in competence, and a choice based on memories of suffering from care. The conclusion is that patient participation in the choice of a healthcare alternative instead of the emergency department is an opportunity of avoiding suffering from care and being objectified.

It is important to make elderly patients’ experiences visible and to underline their rights to influence their choice of healthcare (cf. Dahlberg, Todres, & Galvin, 2009; Enehaug, 2000). This article tries to gain understanding about the healthcare pathway processes implemented for elderly patients, from those who have participated.

## Background

There are challenges to satisfy the patients’ need for security and to provide the best possible care (Benner-Forsberg et al., 2008). To make this possible, the elderly patient should receive optimal healthcare based on her/his healthcare needs. This status highlights questions concerning information given to patients and enabling them to participate in planning for healthcare (SFS, 2010:659), as well as respect for the individual's self-determination and integrity (SFS, 1982:763). The patients’ right to participate is stressed (Fallenberg, 2000). Patient participation is recognized as a key component to improve patient safety (Longtin et al., 2010), and the individual patient and her/his needs are identified as the most essential factor in elderly patients’ participation in the discharge process from hospital (Foss & Askautrud, 2010). Concurrently, patients in general preferred to adopt a passive role (Florin, Ehrenberg, & Ehnfors, 2008). Furthermore, fighting for participation in getting basic needs satisfied score very low in emergency departments (EDs), especially among older and less well-educated patients (Frank, Fridlund, Baigi, & Asp, 2011). Patients with emergency admissions reported lower scores for quality of information than patients with planned admissions (Fröjd, Swenne, Rubertsson, Gunningberg, & Wadensten, 2011). The complexity of patient participation in decision-making is described as a multiple situation factor (Müller-Engelmann, Keller, Donner-Banzhoff, & Krones, 2011) and is affected by various socio-demographic parameters (Eldh, Ekman, & Ehnfors, 2010). Additional predictors for adopting an active participatory role were patients’ gender, education, living condition, and occupational status (Florin et al., 2008).

Among healthcare workers, the acceptance and promotion of patient participation are influenced by the need to maintain control, lack of time, the personnel's beliefs, type of illness, and training in patient–caregiver relationships (Longtin et al., 2010). The individual patient's knowledge was not recognized, and the main focus was from the professional perspective (Frank, Asp, & Dahlberg, 2009). A paternalistic decision was considered most appropriate in emergency situations (Müller-Engelmann et al., 2011).

The interpersonal interaction and exchange between patient and nurse emerged as a central component in the process of participation in nursing care (Petros, Dafni, Chryssoula, Sofia, & Panayota, 2012). Nurse strategies for optimizing patient participation are described as a process of emancipation of the patient's potential by finding her/his own inherent knowledge, values, motivation, and goals. The strategies are important and useful to balance the asymmetry in the nurse–patient relationship in daily nursing practice (Larsson, Sahlsten, Segesten, & Plos, 2009). Patients’ own descriptions of participation focused on having knowledge and interaction with health professionals rather than being informed or partaking in decision-making (Eldh et al., 2010).

The ambulance services have the requested possibility to interact with the elderly patients in nursing care when being responsible for providing opportunities for alternative healthcare pathways (Vicente, Svensson, Wireklint Sundström, Sjöstrand, & Castrén, 2012). Consequently, ambulance personnel have an obligation to interpret and assess patients’ need of healthcare and to invite and involve patients in their choice of healthcare.

The emergency medical services (EMS) have been defined as “Healthcare provided by healthcare professionals within or adjacent to the ambulance” (SOSFS, 2009:10, 2 §) by the National Board of Health and Welfare in 2009. In this study, the term EMS refers to pre-hospital emergency care and the ambulance services.

The aim of this study was to describe patients’ lived experience of participating in the choice of healthcare when being offered an alternative care pathway by the EMS, when the individual patient's medical needs made this choice possible. This research has been carried out within a caring science context (Dahlberg, 2011), which is based on the patient perspective with the general aim to describe care that strengthens and supports health (Dahlberg & Segesten, 2010) and recognizes the patient's suffering as a motivation for care (Eriksson, 2002; Morse, 2001).

## Methods

The study has practised the reflective lifeworld research (RLR) approach as described by Dahlberg, Dahlberg, and Nyström (2008). The method is based on phenomenological epistemology and its lifeworld perspective, that is, understanding human lives, health, suffering, and well-being, as they are experienced in daily life. By adopting the RLR approach, we adopt a reflective stance to that which is taken for granted in everyday life, for example, receiving healthcare from the ambulance service, and describing the phenomenon as it is experienced. The “bridling” of past knowledge is one of the most critical points in this approach. To explore the phenomenon as it is lived, the approach meets the criteria of studying elderly patients’ experiences with openness and sensitivity. We have applied a strategy of being aware of one's own pre-understanding of the phenomenon, to allow the text to present itself without interference from personal beliefs, theories, and other assumptions that might otherwise mislead us and thus prevent us from a new understanding of meaning. Through self-reflection, we endeavored to take control over our preconceptions.

### Setting and sample

All participants (nine female, two male; mean age 81 years) were elderly patients who, based on their medical needs, were included from a randomized study in 2009–2010 (Vicente et al., 2012). Depending on patients’ need of care, the most optimal of three alternatives was presented to patients by the pre-hospital emergency nurse (PEN). The three healthcare alternatives were 1) geriatric ward (GW) (secondary care); 2) community acute care center (CCAC) (primary care); and 3) ED (tertiary care). All informants in this study have chosen GW or CCAC at a community-based hospital (CH) instead of the third alternative, healthcare at an ED.

When these patients were cared for by the EMS, and participated in an alternative healthcare pathway, they also received a request consisting of an invitation to an interview after the healthcare pathway. Furthermore, a week prior to the interview, the research secretary contacted over telephone to make sure that the patients were still interested in participating.

To achieve variation in the data, the participants represent three different periods in relation to the healthcare pathway via the EMS: ≤1 week—three participants, ≥1 month to ≤4 months—three participants, and ≥5 to ≤12 months—five participants. The inclusion criteria for participation in this study, apart from having participated in an alternative healthcare pathway, were that the elderly patient also had to understand the Swedish language and had to be orientated with regard to time and space. Of the 26 patients contacted, 11 agreed to be assigned to this study. No more contacts were taken. The main reasons for refusal were the patient's weakness and frailty.

### Interviews

The interviews were semi-structured (Polit & Beck, 2012) to give the informant the opportunity to recall and reflect on her/his experience in the encounter with the ambulance personnel. The initial question was “How did you experience the healthcare that you received from the ambulance service?” This first question was supplemented by follow-up questions that led the dialog toward patient participation such as “Did you then participate in any way?”, “What did you think about that?”, and “Can you explain it more”, that is, the follow-up questions were dependent upon the informant's answers. The interviews lasted between 40 and 60 min, tape-recorded and subsequently transcribed verbatim. Four interviews took place at the CH, that is, with patients that were still receiving care, and the other seven interviewed in their respective homes.

### Analysis

The analysis followed the phenomenological approach searching for the meaning in accordance with the guidelines of the RLR approach (Dahlberg et al., 2008). This analysis is characterized by a tripartite structure, which can be described as a movement between the whole–the parts–the whole. This is a process of understanding that includes a movement between different abstract levels of meaning.

The analysis started with a reading of the transcript in its entirety, to facilitate an initial understanding. It was a careful reading until the requirement of familiarity with the material was met. When the initial reading was completed, the transcript was slowly re-read and divided into meaningful units—the parts. In these meaningful units, we searched for meanings. Thereafter, the meanings were clustered to uncover similarities and discrepancies in the data. By relating the clusters to each other, a pattern of meanings slowly emerged, which generated a meaningful structure that is the essence of the phenomenon and its constituents. When describing these constituents, the aim was to be truthful to the complexity of data, and consequently the meanings may slightly overlap each other.

The essence of the phenomenon is described in the “Findings” section, followed by descriptions of the meaningful constituents to clarify the meaning further that is, the variations on the essence. Quotations from the participants are included as examples of explicit meanings.

### Ethical considerations

Ethical approval was obtained from the Regional Ethics Committee at the Karolinska Institute, Stockholm (nr. 2008/1167-31). The ethical standards of the Helsinki Declaration (2008) were respected. The PEN obtained informed consent from all participants on the first occasion, when they participated in the choice of an alternative level of healthcare. In connection with the interviews, they received both written and oral information about the purpose of the study, their right to withdraw at any time without prejudicing their cases, and the confidentiality of information given in the interviews.

## Findings

The essence of patient participation in the choice of healthcare, when being offered an alternative care pathway by a carer in the ambulance service, is described as “There was a ray of hope about a caring encounter and about being treated like a unique human being”.

When an acute need for healthcare arises, it reveals vulnerability and fragility in old people's lives. Insufficient insights into one's own health condition conceal the seriousness. A battle for one's independence is fought. That battle turns into a suffering underlined by existential powerlessness. At last, the life-suffering becomes overwhelming and the elderly patients capitulate and express their need for release. A longing for the true rescue starts and survival is associated with the possibility of arriving at a hospital for welcome shelter. Expertise and all necessary treatment are expected to be there, and all hope is focused on getting there as soon as possible. A fear of not immediately getting the right help develops. Suffering is tolerated thinking that hopefully everything will turn out well once the journey to the shelter begins. The elderly patient is then invited to a caring encounter, which is focused on the patient's health condition. A feeling of wellbeing and participation in the choice of healthcare is offered. By keeping close to the patient, the carer offers a feeling of safety and gives the impression of knowing just what the right choice of care is. That choice means that the risks of ending up in the wrong ward and being objectified will hopefully be reduced. After due consideration, the choice of healthcare is finally made and suffering caused by care and the risk of being inhumanely treated are deselected.

The five meaningful constituents are endurable waiting, speedy transference, a concerned encounter, professional competence, and a choice based on memories of suffering from care.

### Endurable waiting

Old people have been shown to practice endurance and to delay seeking care. One man, aged 81, says, “The longer it takes the worse I become, which I've become. I waited too long to call.” The patients make their best effort to try different solutions to self-help as long as it feels meaningful and as long as they have strength left. Waiting is associated with frailty. The old man continues,

I took a lot of medicines, nothing helped. It's a hard thing taking medicines when you're ill. It's hopeless… I think you delude yourself that it'll pass but it doesn't. I'm very frail, at my limit.

Several health problems are often experienced at the same time. With time, the situation becomes even further exacerbated. The elderly patient loses control over her/his life situation. Endurable waiting turns into difficulties with understanding contexts. One 71-year-old woman says,

I think it's hopeless again because I can't take it, not mentally either. I don't know when it happened and I didn't know I was in such bad shape. I don't understand. It must have happened over a long time.

When the older person has waited long enough, her/his strength fails. The situation is complicated by existential uncertainty. Another 81-year-old woman says, “Maybe I was a bit worried that all could end, but I thought that things have worked out before so I think it should this time too, but I don't know.” If there are relatives to assist, help is sought sooner, and the waiting time endured by the patient is shortened.

### Speedy transference

The expectation of speedy transference means that the departure to hospital is expected to start as soon as possible. The elderly patients have a notion that the ambulance personnel do not offer healthcare or medical treatment. One man, aged 86, says, “I can't say that I got any help. I thought we should just hurry to the hospital instead”. One woman, aged 95, says, “I didn't get any care in the ambulance, I just lay there.”

Thus, expectations toward the EMS were low. One woman, aged 80, says, “They did what they could. I don't think it was much more than that.” The mission for ambulance care is described in terms of merely transporting elderly people when they can no longer go to hospital by themselves. One woman aged 77 says, “I didn't expect anything from the ambulance, it was just a means of transport from my house to here.”

The 86-year-old man says that in spite of back pain, he did not want any pain relief from the ambulance personnel. His wish to get to hospital quickly overshadowed all other needs. He says, “They just let me be. I didn't want any either, I just wanted to see a doctor or get into hospital.”

### A concerned encounter

It is seen as an expectation among elderly people that their acute healthcare needs should be treated with an open and friendly attitude. One 95-year-old woman says, “My expectations towards the ambulance personnel are that … they are friendly and nice and quiet …” Encounters like this are characterized as a close relation and understood as supporting the patient's health processes. A relationship involving engagement affects the patient. One woman, soon to be 90, says, “I thought that now I was in good hands. When I'm in good hands I feel safe and taken care of.”

To convey concern, an interpersonal relationship is required with a PEN who is perceived as being present and committed. Such a relationship can make a deep impression on elderly patients. One 95-year-old woman says, “I felt taken care of by the ambulance personnel, I really did. They took me seriously and made me feel safe.”

When an elderly patient experiences an encounter that is concerned, emotions and thoughts are involved which convey sincerity so that the care received is the best at hand. One 81-year-old man says, “I can only say that things couldn't have gone any better.”

### Professional competence

The EMS are experienced by elderly patients as offering healthcare by carers with professional competence whose important mission is to assess elderly patients’ need of healthcare, thus making a unique prioritized assessment based on the needs of the individual. The PEN is experienced at making advanced assessments of where patients with different conditions should be treated and cared for. One woman, aged 71, says,

It's really important that the ambulance personnel are competent! It's they who decide where you need to go, if you should go into the ED for chest pain, stroke or such. The ambulance personnel can decide this.

Professional competence is the foundation for healthcare decisions based on different signs of disease, which include objective signs such as blood pressure as well as subjective signs that only the patients can convey. One woman, aged 75, says that the ambulance personnel observed that “There was no need to hurry with me, it wasn't that critical.” Another assessment is based on the patient's medical history with previous diagnoses. Clear objective signs govern the current assessment. One man, aged 81, says,

I've had a stroke four times, so my right arm capacity is somewhat reduced. That's probably what they were worried about, I think. The ambulance personnel thought I should definitely go to a hospital [CH].

The importance of the professional assessment is emphasized by the fact that a quick decision might be needed to determine the optimal choice of care. There is an awareness of the seriousness of such situations when things need to happen fast and correctly from the start. One woman, aged 75, says:

I think it's a bit scary if it was like that, for someone … if it is something that needs to be done immediately and … it would be terrible if you didn't get to an emergency hospital. But of course, the ambulance personnel are well trained.

### A choice based on memories of suffering from care

Having the opportunity to make a choice of healthcare emerges as a positive invitation to participation, which had never happened before. Previous experiences from emergency care have meant traditional healthcare at an ED. It emerged that old people have memories from healthcare at an ED, which might have been medically insufficient as well as impersonal. Their experiences are perceived as being fragmented into an incompetent context with several doctors involved and without continuity in terms of assessment. One 71-year-old woman says, “Once I saw three doctors before they had decided which unit I should go to.” The ED is experienced as lacking in encounters with concerned carers. Instead, elderly patients encounter a healthcare that is associated with stressed personnel. Their experiences testify to a lack of both caring and medical decisions about what action to take.

Being in need of care without being able to influence your own situation can expose the elderly patient to suffering from care. This is characterized by powerlessness, which is particularly prominent when the patient's lifeworld is not taken into consideration and the caring situation infringes upon fundamental human needs. The patient's experiences are belittled and she/he does not have any way whatsoever to control her/his situation. One 75-year-old woman says, “I wonder when I'll get something to eat, do they know which medicines I'm on? Can I get my medicines?”

Healthcare situations that require a change of environment several times do not address the need to be seen as a human being and getting the feeling of “being somebody”. In cases where patients are unable to experience such feelings, they experience instead a suffering from care characterized by feeling like an object being pushed around. Situations like these are dominated by a hospital's lack of structure and logistics. One 75-year-old woman says,

I'm being pushed around and I don't know if anyone cares. Maybe it's a childish wish that someone will care about me? It's probably a childish wish.

Elderly patients’ encounter with healthcare at the geriatric ward [GW] is different. Not having to wait for care is one experience that is particularly prominent. One 77-year-old woman says,

Here [GW] somehow you get in and you get a bed and it's all taken care of, everything goes much more smoothly … They [the personnel at the GW] come whenever I need help.

At the geriatric ward, the encounter with the carer is experienced as helping the patient to feel like a unique human being, offering healthcare that assists her/him to get back to life. There is a focus on the patient's lifeworld. With the aim of supporting and strengthening the individual's health processes, geriatric healthcare takes the individual patient's life situation as its point of departure. One 77-year-old woman says:

I came into the GW at night. I saw a female doctor and she had a specialist there. They took tests and gave me pills right away … So in the morning the physical therapist takes care of me, so now I can walk on my legs again. I didn't think I'd be able to do that. So from that point of view, I'm much calmer … Here at the geriatric ward the people who take care of you are so good and you can become mobile again, they're very careful about that.

## Discussion

This study discovered the complexity of the elderly patients’ lived experiences when being offered an alternative healthcare pathway, instead of traditional emergency healthcare at the ED. Their choice of healthcare was based on their previous experiences of emergency healthcare as well as in confidence in the PENs’ advanced assessment and recommendations.

The findings reveal that the meaning of the phenomenon is to choose healthcare where there is hope of being treated like a unique human being. If not, the elderly person in need of help will think of herself/himself as a “thing” which can be pushed around in the ED not knowing if anyone cares. This study highlights the value of the fact that experience of illness and disease, in the caring science perspective, involves embodied experiences, as described by Merleau-Ponty (2002). How the elderly gain access to the world and live with their suffering is made apparent, that is, the life situation and feelings that come with illness and disease in old age are expressed through the elderly body. In agreement with Dahlberg (2011) and Wireklint Sundström and Dahlberg (2011), disease involves a subjective dimension of health and the importance of not reducing the patient to her/his symptoms or making her/him into an object is underlined.

According to Gadamer (1993/1996), health is silent and when illness disturbs our life we are no longer free to participate in everyday activities and our life gets limited. Consequently, this study shows how elderly patients’ vulnerability results in entering into total dependency on healthcare, when health is failing. Disease in its more severe forms always reveals the helplessness and fragility of life. This is one of the main reasons why elderly patients chose to go to a CH instead of to the ED, that is, they distanced themselves from being treated inhumanely. Complementing the findings of Dahlberg et al. (2009) and Berglund, Westin, Svanström, and Sundler (2012), we therefore strongly suggested the application of a lifeworld-led care approach, which is more than merely patient-led care. With such an approach, we meet every patient as a unique individual with her/his own experiences of illness and not just as a diagnosis or as a category of patients. All kinds of generalization in treatment and care are then impossible. The values in the lifeworld-led care approach lead to people always feeling valued by the health service and treated with respect, dignity, and compassion (Dahlberg et al., 2009). Based on the present findings, we argue that the healthcare of elderly people must be lifeworld-centered both in the EMS and the ED as well as in the CH. With this caring approach, the human being is understood as being unique and is the foremost expert on herself/himself, her/his suffering and wellbeing, and her/his life.

The findings in this study made “old” experiences of suffering from care visible. Such suffering is, in a caring science perspective, unnecessary and meaningless and a result of healthcare actions that neglected the patients’ perspective and experiences (Arman, Rehnfelds, Lindholm, Hamrin, & Eriksson, 2004; Nåden & Eriksson, 2004). Suffering from care has its origins in the care relationship or in the circumstances of care, such as medical errors or time delays, which create feelings of powerlessness. Consequently, a feeling of exclusion is born, which in turn results in lack of opportunity for elderly patients to be involved in their own care. Complementing the findings of Berglund et al. (2012), we therefore argue that suffering from care needs to be understood as being caused by barriers to patient participation.

Complementing the findings of Nyström, Dahlberg, and Carlsson (2002), we found a lack of a holistic perspective. The elderly patients experienced the absence of holistic nursing care in the ED, and they were abandoned to the arbitrariness of the carer when it came to willingness to provide care. This has been expressed as anxiety about the everyday routines that must be carried out regardless of other circumstances. The patients’ anxieties about needing something to eat and drink, about routine medication, and other daily and fundamental human needs emerged, that is, patients’ powerlessness was made visible. Furthermore, the findings in this study show how these feelings of powerlessness result in elderly patients’ growing resignation. Thus, when an elderly patient has to lie waiting for a long time to be attended to, she/he will probably finally avoid asking the emergency personnel for help with her/his daily routine tasks. As Sahlsten, Larsson, Sjöström, Lindencrona, and Plos (2005) note, one hindrance for patient participation in nursing practice is shortcomings in building a trustful relationship. They discuss lack of insight, lack of knowledge, and a paternalistic attitude as such shortcomings. Therefore, it is crucial that an interpersonal encounter develops, making it possible for patients to express their needs. According to Nyström et al. (2002) and Wireklint Sundström and Dahlberg (2011), nursing practices need to account for patients’ contribution in care and decision-making processes. Consequently, and based on the present findings, we argue that healthcare must be lifeworld-centered with special carefulness toward vulnerable old people.

The findings underline how vital it is not to infringe on the integrity of the patient. Therefore, it is essential to avoid her/his becoming merely a spectator in her/his own care context, since this runs counter to the central goals of the healthcare service (SFS, 1982:763). However, our findings show that patient participation is still abstract and complex, despite extensive implementation. From a caring science perspective, patient participation means that the patient receives information individually which aims to encourage and empower the patient to become involved in her/his own healthcare and which requires focus on the interpersonal relationship between the patient and the carer (The Swedish Association for Ambulance Nurses, 2012). This highlights the fact that the carer must create a caring atmosphere that enables the patient to be met as a human being. Complementing the findings of Eldh (2006), Sahlsten et al. (2007), and Johansson, Ekwall, and Wihlborg (2011), it is strongly suggested that patient participation is about an admission of responsibility but also about gaining a deeper understanding of her/his medical situation.

The findings showed that the ambulance was seen only as a simple means of transport for quick access to healthcare [hospital]. When an elderly person realizes that her/his self-help is no longer sufficient, it creates anxiety and insecurity. Their fear makes them immediately want to go to hospital for help, as uncertainty about their survival grows in intensity (Bowman, 2001). An ambulance is requested when the patients are incapable of making the journey on their own and the ambulance arrival is perceived as a guarantee of getting to hospital (Ahl & Nyström, 2012).

Furthermore, a paradox emerged in this study that showed that the elderly had low expectations concerning the EMS on the one hand, and on the other hand they had high expectations regarding the PENs’ competence to make healthcare decisions. The low expectations may mirror the fact that the participants were patients who were not in need of the expertise of emergency medicine in the ED. Other underlying causes could be that this stands for the traditional attitudes toward the EMS. However, these findings also demonstrate that the elderly experienced that they were invited to a meaningful caring encounter experiencing they were “in good hands”. The elderly patients felt that they were the center of the PENs’ attention, which in turn led to a feeling of receiving good and secure healthcare. This could be understood as one aim with a caring encounter was reached, i.e. that the help-seeker has the right to expect respect and help, which Svenaeus (2003) expresses as disease evoking the carers’ responsibility. In this study, it is shown that when elderly patients were given the option of choosing healthcare performed by ambulance personnel, the patients saw this approach as a positive invitation of help. Thus, it is shown that the elderly relied on the PENs’ assignment of care alternatives, even though they could see the potential risk in a recommendation proving to be wrong. The elderly patient reflected and weighed the risks. Their memories from the ED, of unnecessary suffering, made them reject having to subject themselves to similar humiliations.

In agreement with Melby and Ryan (2005), and based on the present findings, it is strongly suggested that the ambulance care of elderly patients includes holistic assessment and care. This means that the carer, in her/his special assignments, is responsible by virtue of her/his medical and nursing knowledge and skills to enhance and promote the patient's health and well-being.

Finally, our findings show that the elderly patients’ choice of healthcare turned out to be the right decision for the individual and they experienced that their choice fulfilled their care needs. According to Dahlberg et al. (2009), it could be understood as lifeworld-led healthcare when the elderly expressed that they felt respected by and in the carer's focus. Such a caring approach led to the elderly feeling in control of their situation. The patients’ integrity was respected—that increased patient participation—by creating an atmosphere where elderly patients gained courage to express both their need for care and their basic human needs. This is clarified by one elderly patient's experiences of the carer coming to her when she needed help. This response resulted in a feeling of being treated as a unique human being.

Additional research studies are needed to understand better how to prevent suffering from care in elderly people while under emergency healthcare. Furthermore, it is clearly necessary to gain deeper knowledge of how to increase elderly patients’ awareness of the EMS’ possibilities and potential regarding both medical and nursing care.

### Limitations and trustworthiness

The aim of the study implies that only those patients that selected an alternative to ED were interviewed. The patients that chose the ED were not interviewed. It could be seen as a selective sample of participants and is therefore a study limitation.

The trustworthiness of the study was reinforced by the fact that two authors (VV, BWS) read the interviews independently of each other. The meanings were clustered into a meaningful structure by the two authors and reflected on by the whole research team. This multi-professional team is a further guarantee for the analysis that has been carried out. Furthermore, the first author (VV) works as a PEN and has experience of taking care of elderly people in the EMS.

The RLR approach (Dahlberg et al., 2008) puts great demands on the researcher both in terms of data collection and analysis. There is a strong risk of applying preconceived understanding too soon, when the objective in phenomenological research is to reveal the lived experiences of the participants. Therefore, we practiced one form of endurance throughout the research process by being “uninformed” and “not knowing” during interviews as well as during analysis. This approach involves maintaining openness to what the participants say or indicate, that is, being observant and sensitive to the participants’ world of experiences. However, a qualitative researcher must recognize that one can never completely capture another person's experiences in the same way as the actual person. Conversely, qualitative research has been neglected when it comes to patients’ perspectives in the EMS, perspectives that underpin the value of the findings.

## Conclusions and clinical implications

This study shows that patient participation in the choice of a healthcare alternative instead of the ED provides an opportunity to avoid suffering from care and being objectified. In addition, it is absolutely necessary that ambulance personnel support the elderly patient's influence in the caring relationship, thus enabling her/him to participate in healthcare to the greatest possible degree. This is actually done by persistently asking for the elderly person's experiences of health, illness, and suffering.
